# Association between Extremely Low-Frequency Electromagnetic Fields Occupations and Amyotrophic Lateral Sclerosis: A Meta-Analysis

**DOI:** 10.1371/journal.pone.0048354

**Published:** 2012-11-26

**Authors:** Hongjie Zhou, Guangdi Chen, Chunjing Chen, Yunxian Yu, Zhengping Xu

**Affiliations:** 1 Bioelectromagnetics Laboratory, School of Public Health, Zhejiang University School of Medicine, Hangzhou, China; 2 Department of Epidemiology and Health Statistics, School of Public Health, Zhejiang University School of Medicine, Hangzhou, China; Kagoshima University Graduate School of Medical and Dental Sciences, Japan

## Abstract

**Objectives:**

To estimate the relationship between exposure to extremely low-frequency electromagnetic fields (ELF-EMF) and the risk of amyotrophic lateral sclerosis (ALS) by a meta-analysis.

**Methods:**

Through searching PubMed databases (or manual searching) up to April 2012 using the following keywords: “occupational exposure”, “electromagnetic fields” and “amyotrophic lateral sclerosis” or “motor neuron disease”, seventeen studies were identified as eligible for this meta-analysis. The associations between ELF-EMF exposure and the ALS risk were estimated based on study design (case-control or cohort study), and ELF-EMF exposure level assessment (job title or job-exposure matrix). The heterogeneity across the studies was tested, as was publication bias.

**Results:**

Occupational exposure to ELF-EMF was significantly associated with increased risk of ALS in pooled studies (RR = 1.29, 95%CI = 1.02–1.62), and case-control studies (OR = 1.39, 95%CI = 1.05–1.84), but not cohort studies (RR = 1.16, 95% CI = 0.80–1.69). In sub-analyses, similar significant associations were found when the exposure level was defined by the job title, but not the job-exposure matrix. In addition, significant associations between occupational exposure to ELF-EMF and increased risk of ALS were found in studies of subjects who were clinically diagnosed but not those based on the death certificate. Moderate heterogeneity was observed in all analyses.

**Conclusions:**

Our data suggest a slight but significant ALS risk increase among those with job titles related to relatively high levels of ELF-EMF exposure. Since the magnitude of estimated RR was relatively small, we cannot deny the possibility of potential biases at work. Electrical shocks or other unidentified variables associated with electrical occupations, rather than magnetic-field exposure, may be responsible for the observed associations with ALS.

## Introduction

Amyotrophic lateral sclerosis (ALS) is a devastating neurodegenerative disorder that results in the loss of motor neurons, and a rapidly progressive and fatal muscle paralysis. Although some ALS cases are familial, about 90% are sporadic [1]. ALS is considered to be a multifactorial disease with environmental and genetic risk factors [2], [3]. Epidemiological studies have suggested that exposure to various agents in the workplace, such as lead, aluminum, pesticides, electromagnetic fields (EMF), and electrical shocks, is associated with motor neuron degeneration [4], [5], [6], [7].

Studies over the past two decades have shown that occupational exposure to extremely low-frequency EMF (ELF-EMF) may be a causal factor of ALS [6]. ELF-EMF have frequencies ranging from 3 Hz to 3,000 Hz. Workers who are exposed to ELF-EMF include electric power installers and repairers, power plant operators, electricians, electrical fitters, electrical and electronic equipment repairers, train drivers, telephone installers and repairers, and persons operating electrical equipment such as welders, carpenters, and machinists. In 1986, Deapen and Henderson first reported an increased risk of ALS among people with occupations related to electricity and electronics [8]. Since then, numerous epidemiological studies have been conducted to investigate the effect of occupational ELF-EMF exposure on the development of this disorder [9]. Overall, studies in the 1990s consistently reported an association between occupational ELF-EMF exposure and ALS risk; however, the relevant reports in the 2000s are controversial. Some studies found a positive association between occupational ELF-EMF exposure and ALS risk [10], [11], some studies reported an increased ALS risk associated with electrical occupations but not ELF-EMF exposure [12], [13] and others did not indicate associations[14], [15], [16]. Thus, the relationship between ELF-EMF exposure and increased risk of ALS is still questionable in the occupationally-exposed population.

The inconsistent results across the studies may be due to the small sample size in each study, different study design and measurement of ELF-EMF exposure level. To fully evaluate the association between occupational ELF-EMF exposure and ALS risk, we systemically reviewed all published papers and performed a meta-analysis by pooled analyses of all studies and sub-analyses based on study design, exposure assessment method and criteria for ALS diagnosis.

## Materials and Methods

### Literature search methods

This systematic review focuses on the association between occupational exposure to ELF-EMF and ALS risk among the exposed population. We conducted a literature search in the PubMed database up to April 2012 using the following keywords: “occupational exposure”, “electromagnetic fields” and “amyotrophic lateral sclerosis” or “motor neuron disease”. Additional studies were identified by manual search from the references of original studies or review articles on this topic. Full texts or abstracts of all related reports were then reviewed. The literature retrieval was performed by three independent reviewers (H Zhou, G Chen and C Chen).

### Selection criteria

The selected studies were required to meet all the following criteria: (1) each included study must be an unrelated case-control or cohort study and only the one with a larger sample size was selected if studies had partly overlapping subjects; (2) the studies should refer to the association between occupational ELF-EMF exposure and ALS risk; and (3) the outcome should be defined as a medical diagnosis of ALS or registered as ALS on the death certificate.

### Data extraction

Occupational exposure to ELF-EMF is defined as workers exposed to ELF-EMF during the working period, such as electric power installers and repairers, power plant operators, electricians, electrical fitters, electrical and electronic equipment repairers, train drivers, telephone installers and repairers, and workers operating electrical equipment such as welders, carpenters, or machinists. In this meta-analysis, the exposure level of ELF-EMF was classified by job title or job-exposure matrix. The exposure level was assessed according to job title, then workers were divided into two categories: “electrical occupations” (exposure group) and “non-electrical occupations” (no-exposure group). Exposure level to ELF-EMF was measured by the job-exposure matrix, and then the exposure level was expressed as quantitative data. We extracted the relative risk (RR)/odds ratio (OR) and 95% confidence interval (95%CI) of risk of ALS and ELF-EMF exposure from the literature. However, if ELF-EMF exposure was estimated by the job-exposure matrix, only the OR/RR and 95%CI of the highest exposure group was extracted for final analyses.

ALS classification was based on clinical diagnosis or the death certificate. The criteria for ALS diagnosis followed the International Classification of Disease (ICD-8 348, ICD-9 335.2 and ICD-10 G12.2) and the World Federation of Neurology El Escorial.

The following information was extracted from the selected publications: first author, year of publication, study population, study design, duration of case retrieval or cohort establishment, method of case ascertainment, exposure assessment criteria, confounding variables, main results, and quality assessment of studies.

### Meta-analysis

All analyses were conducted with Stata Software, version 11.0 [17]. First, heterogeneity between studies was assessed by χ^2^-based Q-tests and I^2^ tests. A significant amount of heterogeneity was detected if the χ^2^ test was significant. I^2^ values range between 0% and 100% with values of 25%, 50% and 75% interpreted as indicating low, moderate and high heterogeneity. Therefore, if the heterogeneity value was non-significant, the fixed-effect model was used for analyses; otherwise, if the I^2^ values were moderate to high, the random-effect model was applied. The RR/OR and 95%CI were calculated to assess the risk of ALS from exposure to ELF-EMF. To estimate the accuracy and stability of the pooled effect size, separate meta-analyses were performed based on study design (case-control *versus* cohort), criteria for ALS diagnosis, or method of exposure assessment. ALS incidence rate is ∼1.89 per 100,000/year [18]. So the odds ratio (OR) of ALS in case-control studies approximates relative risk (RR). Therefore, when an association between ELF-EMF and the risk of ALS was estimated in analyses of the pooled studies, the OR was used as an RR index. Publication bias was assessed graphically by funnel plots and formally by both Begg's test [19] and Egger's test [17]. A funnel plot allows evaluation of possible publication bias by examining the distribution of the effect size of the OR. The statistically significant level was 0.05.

## Results

### Eligible studies

**Figure 1:**
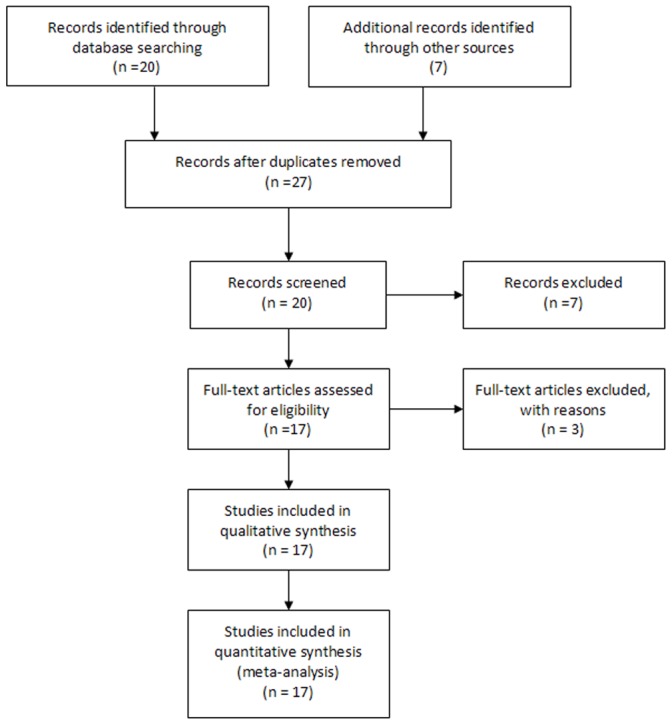
Flow diagram of studies identification.

Twenty abstracts were retrieved, and ten studies were identified as eligible. Out of the twenty, one study was excluded since it was an animal study [20], six articles were review papers [9], [21], [22], [23], [24], [25], and three studies were excluded due to partly overlapping subjects or were case reports [26], [27], [28]. Additionally, we included other seven eligible studies after manual searching from the references of original studies and review articles, or citations from the sources in the “Web of Knowledge” [7], [8], [15], [29], [30], [31], [32]. Finally, seventeen studies meeting the inclusion criteria were identified as eligible (Figure 1). Table 1 shows the characteristics of the studies included for this meta-analysis. Among the seventeen, nine were case-control studies [7], [8], [12], [14], [29], [30], [31], [33], [34] and eight were cohort studies [10], [11], [13], [15], [16], [32], [35], [36]. Seven studies assessed ELF-EMF exposure levels based on job title [7], [8], [15], [29], [30], [31], [34], five were based on the job-exposure matrix [10], [11], [14], [33], [36], and five were based on both [12], [13], [16], [32], [35]. For ALS diagnosis, six studies used clinical diagnosis [7], [8], [10], [30], [31], [33], and the others used the death certificate [11], [12], [13], [14], [15], [16], [29], [32], [34], [35], [36].

**Table 1:** Characteristics of epidemiological studies of the association between occupational exposure to ELF-EMF and ALS risk.

First author, year	Study population (country)	Design	Time period	Method of case ascertainment	Confounding variables	Exposure assessment, criteria	Main results
Deapen, 1986 [8]	518 cases and 518 controls (USA)	Case-control	1977–1979	Clinical examination	Age, sex	Questionnaire survey by mailJob title	OR = 3.8 (1.4–13.0)
Gunnarsson, 1991 [29]	1,961 cases and 2,245 controls (Sweden)	Case-control	1970–1983	Death certificates	Age, sex	1960 national censusJob title	OR = 1.5 (0.9–2.6)
Gunnarsson, 1992 [30]	92 cases and 372 controls (Sweden)	Case-control	1960–1990	Clinical examination	Age, sex	Questionnaire survey by mailJob title	OR = 6.7 (1.0–32.1)
Strickland, 1996 [31]	25 cases and 50 controls (USA)	Case-control	1982–1992	Clinical examination	Age, sex, residence, physical capacities	InterviewJob title	OR = 8.0 (0.9–72.0)
Davanipour, 1997 [33]	28 cases and 32 controls (USA)	Case-control	Not mentioned	Clinical examination	Age, sex, education, socioeconomic status	Questionnaire by interview Job-exposure matrix	OR = 2.3 (0.8–6.6)
Savitz, 1998a [34]	114 cases and 228 controls (USA) nested in occupational cohorts	Case-control	1985–1991	Death certificates	Age, calendar year, social class, men only	From death certificateJob title	OR = 1.3 (1.1–1.6)
Savitz, 1998b [35]	Cohort of 139,905 men and 33 cases (USA)	Cohort	1950–1986	Death certificates	Age, calendar year, race, social class, work status, PCB exposure, solvent exposure	Occupational recordsJob titleJob-exposure matrix(>1.1 µT)	RR = 2.4 (0.8–6.7)RR = 1.2 (0.5–3.0)
Johansen, 2000 [10]	Cohort of 30,631 persons and 20 cases (Denmark)	Cohort	1978–1993	Clinical examination	Age, calendar period, duration of employment	Job-exposure matrix(>1.0 µT)	RR = 1.56 (0.29-8.53)
Noonan, 2002 [12]	312 cases and 1,248 controls (USA)	Case-control	1987–1996	Death certificates	Age, race, social class, men only	Death certificatesJob titleJob-exposure matrix(>0.3 µT)	OR = 2.3 (1.29–4.09)OR = 0.77 (0.37–1.59)
Feychting, 2003 [13]	Cohort of 4,812,646 persons and 1965 cases (Sweden)	Cohort	1981–1995	Death certificates	Age, sex, social class	1970 and 1980 censusesJob titleJob-exposure matrix(>0.5 µT)	RR = 1.4 (1.0–1.8)RR = 0.7 (0.6–1.0)
Hakansson, 2003 [11]	Cohort of 537,692 men and 180,529 women and 97 cases (Sweden)	Cohort	1985–1996	Death certificates	Age, sex, social class	Job-exposure matrix(>0.5 µT)	RR = 2.16 (1.01–4.66)
Weisskopf, 2005 [15]	Cohort of 1,184,561 persons and 937 cases (USA)	Cohort	1989–2002	Death certificates	Age, sex	Questionnaire by interviewJob title	RR = 0.99 (0.49–1.99)
Park, 2005 [14]	6347 cases (USA)	Case-control	1992–1998;	Death certificates	Age, sex, race, region, socioeconomic status	Job-exposure matrix(0.9–0.99 µT)	OR = 0.94 (0.73–1.20)
Roosli, 2007 [16]	Cohort of 20,141 persons and 15 cases (Switzerland)	Cohort	1972–2002	Death certificates	Age, time period, men only	death certificatesJob titleJob-exposure matrixCumulative lifetime Exposure >median	RR = 1.31 (0.31–5.59)RR = 2.32 (0.70–7.73)
Sorahan, 2007 [32]	Cohort of 79,972 persons and 68 cases (England)	Cohort	1973–2004	Death certificates	Age, sex, socioeconomic status	Occupational recordsJob titleEstimated cumulative exposure to magnetic fieldsCumulative year >20 µT	RR = 0.87 (0.67–1.10)RR = 1.45 (0.60–3.55)
Fang, 2009 [7]	109 cases and 253 controls (England)	Case-control	1993–1996	Clinical examination	sex, age	InterviewJob title	OR = 1.4 (0.9–2.3)
Parlett, 2011 [36]	Cohort of 307,012 persons and 40 cases (USA)	Cohort	1979–2011	Death certificates	age, sex, and education	Job-exposure matrix>0.27 µT	RR = 0.98 (0.39–2.50)

### Quantitative synthesis

**Figure 2:**
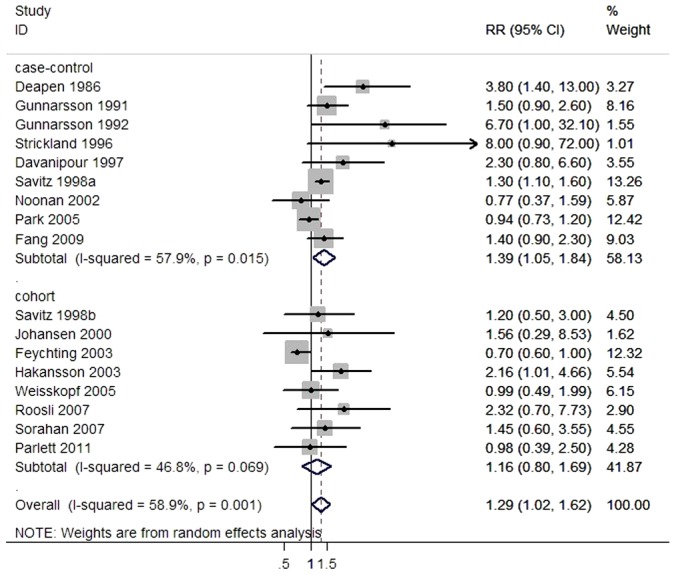
Forest plot of the association between ALS risk and occupational exposure to ELF-EMF.

The main results of this meta-analysis and the heterogeneity test are shown in Table 2 and Figure 2. The data demonstrated that occupational exposure to ELF-EMF was associated with a mildly increased risk of ALS in the pooled studies (RR = 1.29, 95%CI = 1.02–1.62) and case-control studies (OR = 1.39, 95%CI = 1.05–1.84), but not the cohort studies (RR = 1.16, 95%CI = 0.80–1.69). When the ELF-EMF exposure level was evaluated by job-title, the exposure group had a higher risk of ALS in pooled studies (OR = 1.45, 95% CI = 1.15–1.84) and case-control studies (OR = 1.76, 95% CI = 1.27–2.44), but not in cohort studies (OR = 1.16, 95% CI = 0.83–1.61). However, when assessed by the job-exposure matrix, the ELF-EMF exposure was not significantly associated with a risk of ALS, irrespective of the kind of study design.

**Table 2:** Pooled estimates of the association between occupational exposure to ELF-EMF and ALS risk in pooled analyses and separate analyses.

Subgroup analysis	Case-control studies			Cohort studies			Pooled studies		
	No.*	OR(95% CI)	I^2^ (*P*)†	No.*	RR(95% CI)	I^2^ (*P*)†	No.*	RR(95% CI)	I^2^ (*P*)†
**All**	9	1.39(1.05–1.84)	57.9% (0.015)	8	1.16 (0.80–1.69)	46.8% (0.069)	17	1.29(1.02–1.62)	58.9% (0.001)
**Exposure assessment method**									
Job title	7	1.76 (1.27–2.44)	50.0% (0.062)	5	1.16 (0.83–1.61)	51.3% (0.084)	12	1.45 (1.15–1.84)	57.5% (0.007)
quantitative data	3	1.01 (0.67–1.52)	33.6% (0.222)	7	1.23(0.79–1.93)	54.3% (0.041)	10	1.09(0.82–1.43)	45.2% (0.059)
**Type of diagnosis**									
Clinical diagnosis	5	2.58 (1.35–4.92)	42.2% (0.140)	1	1.56 (0.29–8.53)	–	6	2.31 (1.34–3.99)	28.3% (0.223)
Death certificate	4	1.13 (0.88–1.45)	52.3% (0.098)	7	1.15 (0.78–1.72)	52.8% (0.048)	11	1.11 (0.88–1.39)	57.5% (0.009)

* Number of studies.

† Percentage of total variation across studies attributable to statistical heterogeneity rather than to chance (25%, low; 50%, moderate; 75%, high); *P* value for heterogeneity test.

In addition, the studies were divided into two types by ALS ascertainment based on the clinical diagnosis or death certificate. The analyses revealed that exposure level was associated with an increased risk of ALS in the studies using clinical diagnosis (RR = 2.31, 95% CI = 1.34–3.99 for all studies, and OR = 2.58, 95% CI = 1.35–4.92 for case-control studies), but not in studies using the death certificate (RR = 1.11, 95% CI = 0.88–1.39). Meanwhile, moderate heterogeneity was present in all analyses.

### Diagnosis of publication biases

**Figure 3:**
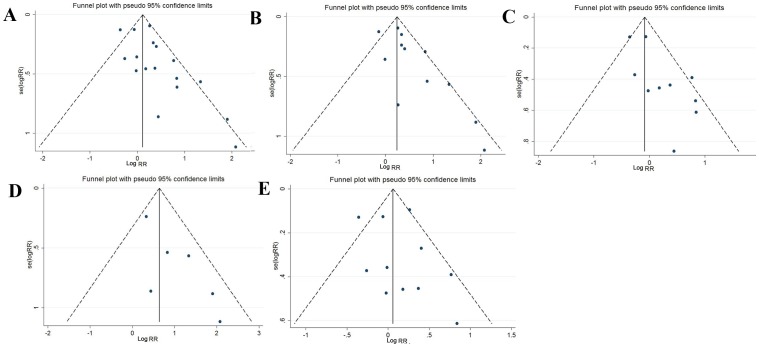
Funnel plot analysis to detect publication bias. Each point represents a separate study for the indicated association. **A** Funnel plot for all studies; **B** funnel plot for job title data; **C** funnel plot for quantitative data; **D** funnel plot for clinical examination data; **E** funnel plot for death certificate data.

Funnel plots were constructed, and Egger's test was performed to assess the publication bias of the selected studies. The shape of Begg's funnel plots revealed marked asymmetry for all effects (Figure 3). These results were confirmed by Egger's test (for pooled studies, *P* = 0.034; for case-control studies, *P* = 0.069; for cohort studies, *P* = 0.009; for job title, *P* = 0.028; and for job-exposure matrix, *P* = 0.020; for clinical diagnosis, *P* = 0.039; for death certificate, *P* = 0.519).

## Discussion

We conducted a meta-analysis of seventeen epidemiological studies on the association between occupational exposure to ELF-EMF and the risk of ALS. The results revealed a slight but significant increase in the risk of ALS among ELF-EMF-related occupations in pooled studies, job-title analysis and clinically diagnosed ALS studies, but not in job-exposure matrix studies and studies of ALS based on the death certificate. Moderate statistical heterogeneity across studies was found in all analyses.

Studies based on job-title showed that electrical occupations increased the risk of ALS, but the result from studies estimating exposure levels of ELF-EMF by the job-exposure matrix suggested that ELF-EMF was not significantly associated with ALS risk. Persons in electrical occupations may have a greater potential for electrical shocks. Electrical shocks or other unidentified variables associated with electrical occupations, rather than magnetic-field exposure, may distort real association between ELF-EMF and ALS risk [12]. If electrical shocks account for the increased risk in electrical occupations, then the highest risk should be expected among electricians; however, current studies do not indicate this association [13]. On the other hand, it has been suggested that exposure to polychlorinated biphenyls may be associated with ALS, and employees in the electric industries may historically have been exposed to these agents in insulating fluids [5], [32]. The possible confounders of co-exposures to these agents should be excluded in future studies.

A significant association was found in the studies of clinically diagnosed ALS, but not in those based on the death certificate. Actually, the analyses of clinically diagnosed ALS studies included four job-title studies [7], [8], [30], [31] and two job-exposure matrix studies [10], [33]. However, the analyses of studies of ALS based on the death certificate comprised eight job-title studies [12], [13], [15], [16], [26], [32], [34], [35] and eight job-exposure matrix studies [11], [12], [13], [14], [16], [32], [35], [36]. Thus, the significant association in clinically diagnosed ALS studies may derive from job-title studies.

However, epidemiological studies have several weaknesses, mostly in relation to case ascertainment and controls selection in case-control studies, exposure assessment, and control of confounders. Incomplete ascertainment of cases decrease the statistical power of case-control studies. In this meta-analysis, we found a higher pooled risk by pooling the studies based on clinical examination when compared to that based on death certificates, and observed substantial heterogeneity when the results from both approaches were compared (*P* = 0.026, Data not shown). Out of nine case-control studies, four selected cases based on the death certificate [12], [14], [29], [34], and five recruited cases from clinical patients [7], [8], [30], [31], [33]. One source of variability is the possible misclassification of the disease, and another may relate to the case-finding period and time from the first exposure. ALS has a long latency/survival period [37], and there is a time lag between exposure to ELF-EMF and the manifestation of this disease, so the information bias is inevitable in some studies. Different strategies were applied for control selection in the nine case-control studies: five population-based studies and four from death certificates. Furthermore, the inclusion and exclusion criteria for controls were substantially different across studies. Also, the methods of estimating exposure levels to EMF were various across studies; and a potential confounding effect would be introduced in individual studies. These sources of variation could explain the moderate heterogeneity in the pooled analysis of the case-control studies.

Twelve studies used “job title” to characterize exposure, whereas ten assessed the levels of exposure to ELF-EMF by the job-exposure matrix. In studies using job title, occupations were grouped into “electrical” and “non-electrical” categories, but the criteria for defining “electrical occupation” varied across studies [12], [13], [15], [16], [26], [32], [34], [35]. Cutoff points of exposure were different across studies using job-exposure matrix [11], [12], [13], [14], [16], [32], [35], [36]. However, different definition of exposure to ELF-EMF across studies may introduce various effects on ALS. These variations may contribute to the moderate heterogeneity in all analyses.

Although potential confounders such as age and sex were controlled in most of these studies, other confounders might have derived from unknown and unmeasured variables. One potential confounding factor could be ELF-EMF exposure from non-occupational sources. ELF-EMF are generated by many sources, including power lines, electric transportation systems, and electrical appliances. These non-occupational sources could result in the same level of exposure as occupational sources [38]. Other potential confounding factors may be introduced in epidemiological studies, such as electrical shocks, trauma, and exposure to organic solvents, metals, and agricultural chemicals [39], [40]. People engaged in electric utility occupations frequently experienced electrical shocks, which was one of ALS risk factors. However, it is difficult to assess the separate effects of ELF-EMF exposure and electrical shocks on the risk of ALS [9].

In addition to occupational ELF-EMF exposure, public exposure to environmental ELF-EMF has increased rapidly in the last a few decades. Future studies should examine the association between public ELF-EMF exposure level and ALS incidence to clarify the relation between ELF-EMF exposure and ALS risk.

### Biological mechanisms

Biological mechanisms have been explored to clarify the association between ELF-EMF exposure and ALS risk. Some laboratory studies indicated that *in vitro* exposure to ELF-EMF produces larger quantities of cellular reactive oxygen species [41], [42] and *in vivo* ELF-EMF exposure induces oxidative stress and impairs antioxidant status in rats [43], [44], [45]. Actually, the oxidative stress plays a key role in the development of ALS although the pathogenic processes involved in ALS are complex [46]. Some studies demonstrated that EMF cause DNA strand breaks in brain cells, resulting in apoptosis and necrosis, which may be involved in the relationship between ELF-EMF exposure and ALS risk [47]. However, no study shows a connection between ELF-EMF exposure, oxidative stress/DNA damage in brain cells and ALS development. Animal models were used to assess the possible effects of ELF-EMF on the development of ALS, but the results did not provide the evidence of such a link [20]. Future laboratory studies are required to systemically investigate the possible role of ELF-EMF on the development of ALS under different ELF-EMF exposure conditions.

## Conclusions

This meta-analysis included seventeen studies of the association between ALS risk and ELF-EMF exposure. Although there are potential limitations from study selection bias, exposure misclassification, and the confounding effect of individual studies in this meta-analysis, our data suggest a slight but significant ALS risk increase among those with job titles related to relatively high levels of ELF-EMF exposure. Since the magnitude of estimated RR was relatively small, we cannot deny the possibility of potential biases at work. Electrical shocks or other unidentified variables associated with electrical occupations, rather than magnetic-field exposure, may be responsible for the observed associations with ALS.

## Supporting Information

S1 PRISMA Checklist (DOC)
